# Oncological Outcomes and Postoperative Complications for Localized Soft Tissue Sarcomas of the Extremities and Trunk Wall in Patients Aged 85 Years or Older

**DOI:** 10.3390/cancers17121940

**Published:** 2025-06-11

**Authors:** Kunihiro Ikuta, Tomohisa Sakai, Hiroshi Koike, Takeo Fujito, Hiroshi Urakawa, Yoshihiro Nishida, Shiro Imagama

**Affiliations:** 1 Department of Orthopedic Surgery, Nagoya University Graduate School of Medicine, 65 Tsurumai, Showa, Nagoya 466-8550, Japan; 2 Rare Cancer Center, Nagoya University Hospital, 65 Tsurumai, Showa, Nagoya 466-8550, Japan; 3 Advanced Medicine, Nagoya University Hospital, 65 Tsurumai, Showa, Nagoya 466-8550, Japan; 4 Department of Rehabilitation, Nagoya University Hospital, 65 Tsurumai, Showa, Nagoya 466-8550, Japan

**Keywords:** older age, postoperative complications, soft tissue sarcoma

## Abstract

Objectives: The management of soft tissue sarcoma in patients aged ≥ 85 years remains a clinical dilemma, as evidence to guide treatment decisions in this population is limited. Here, we aimed to compare the clinical characteristics of patients aged ≥ 85 years who underwent surgery with those who did not, to identify factors influencing surgical decision-making. We also assessed the oncological outcomes and postoperative complications in the patients. Methods: We reviewed 37 consecutive patients with localized disease involving either the extremities or trunk wall, with a median age of 89.0 years. No significant differences in demographics or clinical characteristics were observed between patients who underwent surgery and those who received conservative management. Among the 37 patients, 25 underwent surgery with curative intent. Results: The two-year overall survival rate among surgically treated patients was 77%, with local recurrence-free survival and metastasis-free survival rates of 77% and 57%, respectively. Postoperative complications occurred in approximately one-third of the cases. Compared to those without complications, patients with complications were older (*p* = 0.025), had poorer performance status (*p* = 0.017), were likely to have trunk involvement (*p* = 0.01), and had larger tumor sizes (*p* = 0.025). No significant prognostic factors for overall survival were identified. Conclusions: While surgery is not without risk, our results can provide useful information for both physicians and patients to discuss and explain possible outcomes with surgical treatment.

## Simple Summary

Surgical treatment for soft tissue sarcoma in patients aged ≥ 85 remains challenging due to limited evidence. Among 37 patients with localized disease, 25 underwent surgery. Patient and tumor profiles were similar between patients who underwent surgery and those who did not. The two-year overall survival among surgically treated patients was 77%, and approximately one-third experienced complications. Older age, poor performance status, trunk location, and larger tumors were associated with complications. Although no prognostic factors for overall survival were identified, surgical treatment appears reasonable, given its acceptable complication rates and potential survival benefits.

## 1. Introduction

The global increase in cancer incidence among older individuals is a significant concern, with individuals aged ≥ 65 years accounting for more than half of all newly diagnosed cancer cases worldwide [1]. Soft tissue sarcomas (STS), despite having an incidence of <1% of all malignancies, disproportionately affect older adults [2]. Approximately half of STS cases occur in patients aged ≥ 65 years, with 16% observed in those aged 75 to 84 years [2,3,4,5]. A recent analysis of nationwide data on STS in Japan using the population-based National Cancer Registry reported that patients aged 80 years or older accounted for 19.3% of sarcomas arising from soft tissue and skin [6]. As life expectancy continues to increase, the incidence of STS in the older population is expected to rise further.

Wide resection with negative margins remains the standard treatment for localized STS [7]. A previous study reported a 5-year overall survival rate of 43.2% in patients aged 80 years or older who underwent surgical treatment, suggesting that surgery may be applicable even in very old patients [8]. However, in very old patients, surgical decision-making is complicated by multiple comorbidities, diminished physiological reserves, and considerable variability in individual health status [3,4,9,10].

Additionally, data on postoperative complications in this population remain scarce, and predictive tools for assessing surgical risk are inadequate. This lack of evidence complicates the ability of clinicians to provide accurate prognostic information to patients and their families, causing informed decision-making to be challenging. The National Comprehensive Cancer Network guidelines emphasize the importance of shared decision-making based on prognosis, treatment risks, and patient values, with sufficient information exchange among clinicians, patients, and their families for older adults [11]. A survey of 226 older adults with advanced cancer, severe heart failure, or chronic obstructive pulmonary disease showed that most did not choose life-prolonging treatments if they resulted in loss of independence. While 90% opted for treatment when full recovery was expected, only 25% and 11% opted for treatment when severe physical or cognitive impairment was anticipated, respectively [11]. These findings suggest that older adults often prioritize quality of life (QOL) over merely extending survival.

Analyses from the Surveillance, Epidemiology, and End Results registry have shown a decline in surgical interventions for STS in patients aged ≥ 85 years [10]. Even in cases where no metastasis was observed at the first visit, only 76.3% of patients aged ≥ 85 years underwent surgical therapy for STS [9]. This trend may reflect concerns regarding perioperative morbidity and the overall effectiveness of the treatment in this population. However, the absence of robust data hinders the establishment of evidence-based treatment strategies, leaving uncertainties regarding the optimal management of STS in older patients.

At our institution, patients with localized STS undergo wide resection with negative margins and receive standardized postoperative follow-up, regardless of age. However, in clinical practice, surgical decisions are influenced by individual patient factors, and the impact of these decisions on clinical outcomes and postoperative complications in patients aged ≥ 85 years remains unclear.

This study aimed to compare the clinical characteristics of patients aged ≥ 85 years who underwent surgery with those who did not to identify factors influencing surgical decision-making. Additionally, we investigated oncological outcomes and postoperative complications in patients who underwent curative resection to assess the feasibility of surgery in this population.

## 2. Materials and Methods

This study was approved by the Institutional Review Board of our institution (Approval Number: 2015-0358) and conducted in accordance with the Declaration of Helsinki. We retrospectively reviewed the medical records of patients with STS of the trunk wall or extremities who were treated at our institution between 1995 and 2024. The inclusion criteria were (1) aged ≥ 85 years at the time of diagnosis with histologically confirmed STS and (2) diagnosed with localized disease at presentation. Patients who underwent unplanned excisions outside our institution were included. The exclusion criteria were (1) patients with lymph node involvement or distant metastases at diagnosis; (2) those treated with surgery or radiotherapy for palliative purposes; and (3) patients diagnosed with atypical lipomatous tumors, desmoid fibromatosis, and dermatofibrosarcoma protuberans were excluded due to their distinct clinical behavior, low metastatic potential, and different treatment strategies, which could confound survival analysis.

### 2.1. Data Collection and Variables

We collected demographic and clinical data, including age at diagnosis, sex, age-adjusted Charlson comorbidity index (ACCI), Eastern Cooperative Oncology Group performance status (PS), body mass index (BMI), histological grade (classified according to the Fédération Nationale des Centres de Lutte Contre le Cancer system) [12], tumor size, and tumor location. The Charlson comorbidity index (CCI) was used to evaluate the severity of preoperative comorbidities [13]. The ACCI incorporates patient age into the CCI and has been reported to be a superior prognostic indicator compared to the original CCI [14]. In this study, ACCI was calculated by adding 4 points for patients aged 80–89 years and 5 points for those aged ≥ 90 years to the original CCI score. Accordingly, patients aged 85–89 years with malignant tumors and no other comorbidities had an ACCI score of 6, while those aged ≥ 90 years had a minimum score of 7. Patients with additional comorbidities had even higher scores depending on the number and severity of their conditions. Tumor size was assessed as the maximum diameter measured on MRI at the time of presentation. In cases where unplanned excisions were performed, preoperative imaging studies were either unavailable or, if images were obtained, there was a delay in the referral. Therefore, all cases of unplanned excisions were excluded from the tumor size analysis to maintain consistency. The histological grade was categorized as low (grade 1) or high (grades 2 and 3). Surgical margins were classified as R0 (microscopically negative), R1 (microscopically positive), or R2 (macroscopically positive). The American Society of Anesthesiologists (ASA) score was recorded for surgically treated patients.

### 2.2. Treatment and Oncological Outcomes

Treatment variables included type of surgery (limb salvage or amputation), use of (neo) adjuvant radiation therapy, and utilization of flap closure for soft tissue reconstruction (free, pedicled, or local flap). The length of hospital stay was also recorded. Postoperative complications, such as wound dehiscence and infection, were defined as events occurring within 30 days of surgery with Clavien–Dindo grade ≥ II events [15,16]. Chemotherapy was generally indicated for high-grade tumors > 5 cm and located in deep-seated regions. However, chemotherapy was not administered in patients aged ≥ 85 years, owing to concerns regarding frailty and treatment tolerance. Postoperative radiotherapy was administered for R1 or R2 surgical margins. Follow-up evaluations included clinical examinations, chest CT, and MRI of the surgical field every 3–4 months for the first 2 years postoperatively and every 6 months thereafter. Oncological outcomes, including survival, local recurrence, and distant metastasis, were assessed.

### 2.3. Statistical Analysis

Patient survival was estimated using the Kaplan–Meier method, and survival curves were compared using the log-rank test. Fisher’s exact test was used for categorical variables, and the Mann–Whitney U test was used for continuous variables to analyze correlations between clinical variables. Owing to the limited sample size, multivariate analysis was not performed. Statistical significance was set at *p* < 0.05. Follow-up duration was defined as the time from diagnosis to death or the last visit. All statistical analyses were conducted using SPSS version 29.0 for Windows (SPSS Inc., Chicago, IL, USA). Continuous variables were summarized as means ± standard deviations, and categorical variables as counts and percentages.

## 3. Results

### 3.1. Demographic and Clinical Characteristics

**Figure 1:**
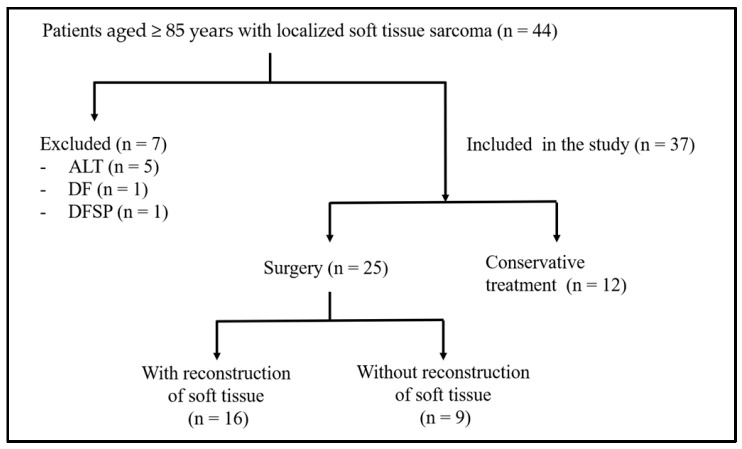
Flowchart of patient selection and treatment. *ALT*, Atypical lipomatous tumor; *DF*, Desmoid fibromatosis; *DFSP*, Dermatofibrosarcoma protuberans.

A total of 37 patients, none of whom had metastatic disease at the time of diagnosis, were included in this study (Figure 1). The mean age at diagnosis was 89.1 ± 3.1 years, and 22 patients (60%) were female. The median PS was 1, and the mean BMI was 21.5 ± 3.6 kg/m^2^. The most common histological subtypes were undifferentiated pleomorphic sarcoma (UPS), myxofibrosarcoma, and dedifferentiated liposarcoma in 16 (43%), eight (22%), and two (5%) patients, respectively. The mean tumor size was 9.9 ± 4.1 cm. The most frequent tumor location was the lower extremities (n = 20, 54%), followed by the trunk (n = 10, 27%) and upper extremities (n = 7, 19%). Histological grading revealed low-grade tumors in seven patients (19%) and high-grade tumors in 30 (81%). Unplanned excision of the tumor at other institutions was noted in 10 patients (27%), four of whom presented with recurrent disease at diagnosis. The mean follow-up duration was 19.4 ± 19.2 months. The patient and tumor characteristics are summarized in Table 1.

### 3.2. Surgical Treatment

Among patients who underwent surgery with curative intent, 68% had an ASA score of ≥II. Excluding one patient with UPS in the thigh who underwent hip disarticulation, limb-salvage surgery was performed in 24 patients (96%). Of these, 20 (80%) achieved R0 margins, four (16%) had R1 margins, and one (4%) had an R2 margin. Soft tissue reconstruction was required in 16 (64%) patients. Flap reconstruction was performed in 11 patients (44%), including free, pedicled, and local skin flaps in three, five, and three patients, respectively. Skin grafting was performed in five patients (20%), whereas nine patients (36%) did not require soft tissue reconstruction. One patient who underwent unplanned excision subsequently received neoadjuvant therapy with carboplatin and etoposide. No patient received postoperative adjuvant chemotherapy. Radiotherapy was administered preoperatively and postoperatively in four (16%) and five (20%) patients, respectively. No patient received both pre- and postoperative radiotherapy. The mean length of postoperative hospital stay for surgically treated patients was 36.3 ± 22.2 days. The mean follow-up period for the surgically treated patients was 23.8 ± 19.6 months. Local recurrence occurred in four patients (16%), and distant metastases were observed in eight (32%), all of whom developed lung metastases as the first site of distant progression. The mean time from diagnosis to initial metastasis was 21.7 ± 20.4 months.

### 3.3. Conservative Treatment

Conservative management was chosen for 12 patients (32%). The reasons for not undergoing surgery included patient or family preference against surgery (n = 8), deterioration of general condition before surgery (n = 2), and insufficient surgical tolerance (n = 2). Among the eight patients who did not wish to undergo surgery, three had been referred after unplanned excision but declined further definitive resection or amputation. Of these, two received additional radiotherapy, and one received best supportive care (BSC). The remaining five patients opted against surgery due to religious reasons (n = 1), concerns about the risk of dialysis (n = 1), absence of family consent (n = 1), preference for radiotherapy in a case of myxoid liposarcoma (n = 1), and inability to accept extensive resection with complex reconstruction for a posterior trunk tumor involving the ribs and spine (n = 1). Ultimately, seven of the twelve patients received radiotherapy, including three who received doses exceeding 60 Gy and one who received proton beam therapy. The other five patients received BSC. There were no significant differences in the baseline variables between patients who underwent surgery and those who received conservative management (Table 1).

### 3.4. Postoperative Complications

A total of 11 postoperative complications with Clavien–Dindo grade ≥ II were observed in eight patients (32%). Among them, five were wound-related complications, including wound dehiscence (n = 2), surgical site infection (n = 2), and flap necrosis (n = 1) (Table 2).

All wound complications occurred in patients who underwent soft tissue reconstruction. Systemic complications were managed with medical therapy. Five complications were classified as Clavien–Dindo grade ≥ III, which required surgical intervention. Four were wound-related. Additionally, one patient developed postoperative pneumothorax, necessitating tracheostomy and mechanical ventilation. No surgery-related deaths were observed.

A comparison between patients with and without postoperative complications revealed that those with extremity tumors had a significantly lower incidence of complications (*p* = 0.01). Patients who developed complications were significantly older (*p* = 0.025), had a higher proportion of PS ≥ 2 (*p* = 0.017), and had larger tumors (*p* = 0.025) than those without complications. No significant difference was observed in the length of hospital stay between the two groups (Table 3).

Our analysis revealed that the type of soft tissue reconstruction was closely associated with postoperative complications. In particular, pedicled flaps were associated with a higher complication rate (83%) compared to other methods. Five of the six patients who underwent pedicled flap reconstruction experienced postoperative complications, including flap necrosis with pneumonia (n = 1), postoperative delirium (n = 1), severe pneumothorax with delirium (n = 1), wound dehiscence (n = 1), and surgical site infection (n = 1). A comparison between patients who underwent primary closure and those requiring any form of soft tissue reconstruction showed a statistically significant increase in postoperative hospital stay in the latter group (*p* = 0.038, Table 4). However, no significant difference was observed in postoperative complication rates between these two groups (*p* = 0.67).

### 3.5. Oncological Outcomes and Prognostic Factors

**Figure 2:**
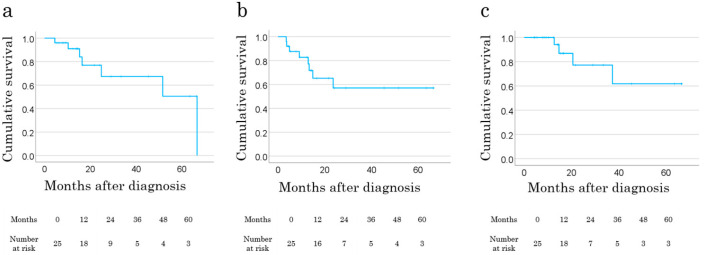
Survival of 25 older patients ≥ 85 years with localized soft tissue sarcomas in the trunk or extremities. (**a**) Overall survival. (**b**) Metastasis-free survival. (**c**) Local recurrence-free survival.

At the latest follow-up, 17 patients (46%) were alive with no disease, 10 (27%) had evidence of disease, eight (22%) had died of disease, and two (5%) had died from other causes, including brain infarction and colon cancer. Among the 25 patients who underwent curative-intent surgery, the 2-year overall survival (OS), metastasis-free survival (MFS), and local recurrence-free survival (LRFS) rates were 77.0%, 57.0%, and 77.2%, respectively (Figure 2a–c). Female sex was associated with improved MFS (*p* = 0.015). However, no clinical factors were significantly associated with LRFS or OS in the univariate analysis. LRFS was comparable between patients with R0 and R1/R2 margins (*p* = 0.98).

## 4. Discussion

The definition of “older” in the context of STS remains inconsistent across the literature, with thresholds ranging from 65 to ≥75 years [2,4,10,17]. This variation complicates comparative analyses, especially when assessing the influence of tumor biology, comorbidities, and functional status on clinical outcomes. Thus, our study focused specifically on patients aged ≥ 85 years, a group excluded from clinical trials and for whom surgical treatment is typically regarded as high risk.

In older patients, the balance between the potential oncologic benefit and surgical risk becomes increasingly delicate. Clinicians may hesitate to recommend surgery due to concerns about frailty, limited life expectancy, and the possibility that complications could significantly reduce postoperative QOL [18]. In our cohort, 68% of patients underwent curative-intent surgery, and no significant baseline differences were observed between those who underwent surgery and those who did not. This may reflect evolving attitudes among clinicians toward offering surgery to selected older patients when their general condition permits and suggests that careful patient selection can broaden the therapeutic window in the older patient population.

Several studies have reported outcomes of surgical treatment in older patients with STS, focusing on patients aged ≥ 80 years. Okamoto et al. reported favorable 2- and 5-year OS rates (84% and 42%, respectively) in patients ≥ 80 years, in both surgically and non-surgically treated patients [9]. Tsuchie et al. investigated patients ≥ 85 years who underwent surgery and reported a 3-year OS of 58% [19], while Basse et al. reported a 2-year OS of 40% in patients ≥ 90 years old [5]. Our 2-year OS of 77% in surgically treated patients aged ≥ 85 years compares favorably with these studies. This suggests that age alone should not be a limiting factor for surgical intervention in appropriately selected patients. However, from a clinical perspective, we must also consider functional independence and postoperative recovery, which often weigh more heavily than survival duration in patient decision-making. Future studies may benefit from incorporating patient-reported outcome measures or gerontological assessments to more accurately assess these aspects.

Wide excision in elderly patients may lead to impaired limb function, potentially resulting in a decline in QOL. However, there remains a paucity of studies specifically addressing postoperative QOL in elderly patients with STS. A recent study using the EuroQol 5-Dimension 5-Level (EQ-5D-5L) in patients aged 70 years or older with malignant bone and soft tissue tumors suggested that appropriate treatment may help preserve postoperative QOL [20]. Patients with better preoperative general health were associated with more favorable postoperative QOL outcomes. Nonetheless, whether to indicate wide excision, which may impair function or to pursue less extensive, function-preserving surgery to maintain QOL remains an unresolved clinical issue in elderly patients with STS. Addressing this question is essential for optimizing their treatment strategies. The rate of wound-related complications and surgical reintervention in our study is consistent with those reported in a meta-analysis by Slump et al., which investigated wound complications following resection of extremity STS (30.2% wound complication rate, 13.4% reoperation rate) [21]. Their study included patients of various age groups, not limited to older adults. All wound-related complications in our cohort occurred in patients who underwent soft tissue reconstruction, suggesting that surgical complexity, rather than age, may be a determinant of postoperative morbidity. Based on our experience, surgical decisions in older patients are influenced more by physiological conditions and the complexity of the planned procedure than by chronological age. Although indicators such as the ASA score are commonly used, our findings suggest that they may not fully reflect the surgical risk specific to older patients. We observed that postoperative complications were more frequent in patients with truncal tumors, advanced age, PS ≥ 2, and larger tumor size. Although soft tissue reconstruction did not significantly affect postoperative complication rates, it was associated with longer hospital stays, potentially increasing overall healthcare costs. Tsuda et al. reported that postoperative complications occurred in 16.8% of patients aged ≥ 65 years, and severe thinness (BMI < 16.0) was a strong risk factor [22]. Slump et al. have identified risk factors for postoperative complications, such as high BMI, diabetes, smoking, and preoperative radiation, which may help guide preoperative risk assessment [21]. We recognize that differences in complication definitions, patient populations, and treatment protocols make it difficult to directly compare risk factors for postoperative complications with those reported in previous studies. Additionally, our relatively small sample size limited our ability to detect statistically significant associations; therefore, our findings should be interpreted cautiously. Nonetheless, the factors associated with postoperative complications in our cohort appear to align with clinical impressions observed in real-world practice for STS in older adults.

Several studies have reported on postoperative complications in elderly patients with cancer. In particular, elderly individuals aged 80 or older undergoing gastrointestinal cancer surgery are at high risk of postoperative complications [23,24,25]. Takeuchi et al. reported that in gastric cancer patients aged ≥ 80 years, the rates of operative mortality and postoperative complications (Clavien–Dindo grade ≥ II) were 1.6% and 50.5%, respectively [23]. They also found that in patients aged ≥ 80 years, pneumonia, delirium, and urinary tract infections were more common compared to those aged < 80 years, with pneumonia and other infections exerting a substantial impact on survival and recovery in the older group. In our cohort of elderly patients undergoing surgery for STS, wound complications, including surgical site infection and delayed healing, were the most frequently observed. This finding is consistent with a previous study by Tsuda et al. [22]. In contrast, systemic complications tend to occur in elderly patients undergoing gastrointestinal cancer surgery. This may reflect the higher physiological burden and systemic stress associated with gastrointestinal cancer surgery compared to soft tissue procedures.

Unlike previous reports, our study did not reveal any significant prognostic factors for OS, including BMI and PS. Tamiya et al. reported poor PS (one or two) and low BMI (≤ 25) as independent predictors of OS in patients with STS aged ≥ 60 years [26]. Tsuda et al. found that sarcoma-specific survival in patients aged ≥ 65 years was significantly worsened by R1 surgical margins, advanced tumor stage, and poor PS (≥ 2) [22]. In our study, however, no significant associations were observed, even for tumor grade and larger size, which are widely recognized as key prognostic factors in STS [27]. The absence of significant prognostic factors for OS in our study may be partly explained by the inclusion of low-grade tumors (20%), which could have reduced the statistical power to detect differences. These results may also reflect the inherent limitations of our study, including a relatively short follow-up period and small sample size, which may have limited the ability to detect longer-term survival trends.

In addition to these factors, several other limitations should be acknowledged. First, despite a small sample size, this study included a heterogeneous group of histological subtypes with diverse biological behaviors, making direct comparisons of the surgical outcomes challenging. Second, as a retrospective study, inherent selection bias and unadjusted confounding factors cannot be excluded. Therefore, caution should be exercised in applying these results to younger patients or those with more advanced stages of STS. Third, formal frailty assessment tools, such as the Geriatric 8 or Clinical Frailty Scale, were not included in this study. Instead, PS and the ACCI were used as surrogate indicators of functional reserve and comorbidity burden. Future prospective studies incorporating standardized frailty assessments or QOL evaluation may provide more accurate stratification and decision-making support in the elderly population. Fourth, we included patients who had undergone unplanned excision. Once a definitive diagnosis was made, these patients were promptly referred to our center, where additional wide resection was performed in a timely manner, as the residual tumor is often found in the initial surgical bed. In fact, all 10 such patients in our cohort underwent re-excision within two months. We believe that including these cases reflects actual clinical practice and supports the clinical relevance of our findings. Lastly, in the conservative treatment cohort, several patients were transferred to other institutions or received home-based care, making it difficult to accurately capture final outcomes or survival data.

## 5. Conclusions

Our results may help inform treatment discussions for patients aged ≥ 85 years with localized STS. The findings suggest that, with appropriate patient selection, surgical treatment can be a practical and effective approach in this very old population, with acceptable complication rates and potential survival benefits. Postoperative complications were more common in patients with poor PS, non-extremity tumor location, and larger tumor size, emphasizing the importance of careful preoperative evaluation. Although the retrospective nature and limited cohort size may limit the strength of general conclusions, our findings can provide practical insights for clinical care in very elderly patients with localized STS, though the results should be interpreted with caution. Further research is needed to guide treatment for the growing population of older adults.

## Figures and Tables

**Table 1:** Demographic data of 37 patients ≥ 85 years with localized soft tissue sarcomas in the trunk and extremities.

Variables	All Patients (n = 37)	Surgically Treated (n = 25)	Conservative Treatment (n = 12)	*p*-Value
Age (years, mean)	89.1	88.6	90.1	0.14
Female	22 (59%)	16 (64%)	6 (50%)	0.49
PS ≥ 2	11 (30%)	7 (28%)	4 (11%)	0.39
BMI (kg/m^2^, mean)	21.5	21.5	21.1	0.48
ACCI ≥ 9	10 (27%)	6 (24%)	4 (33%)	0.45
Extremity tumor	24 (65%)	16 (64%)	8 (67%)	1.00
Diagnosis				0.09
UPS	16	12	4	
MFS	8	7	1	
DDLPS	2	0	2	
Others ^a^	11	6	5	
High-grade tumor	30 (81%)	20 (80%)	10 (83%)	1.00
Size (cm, mean)	9.9	10.1	9.4	0.60
Previous unplanned excision	10 (27%)	7 (28%)	3 (25%)	1.00

^a^: Other diagnoses included pleomorphic liposarcoma (n = 1), extra-skeletal chondromyxoid sarcoma (n = 1), leiomyosarcoma (n = 1), angiosarcoma (n = 1), myxoid liposarcoma (n = 1), fibrosarcoma (n = 1), undifferentiated sarcoma (n = 2), malignant tenosynovial giant cell tumor (n = 1), unknown (n = 2). *PS*, performance status; *BMI*, body mass index; *ACCI*, age-adjusted Charlson comorbidity index; *UPS*, undifferentiated pleomorphic sarcoma; *MFS*, myxofibrosarcoma; *DDLPS*, dedifferentiated liposarcoma.

**Table 2:** Postoperative complications in 25 patients ≥ 85 years with localized soft tissue sarcomas in the trunk and extremities.

Variables	n	Clavien–Dindo Grade	
		II	≥III
Wound related complication			
Wound dehiscence	2	0	2
Wound infection	2	1	1
Flap necrosis	1	0	1
Systemic complication			
Pneumonia	1	1	0
Bacteremia	1	1	0
Pneumothorax	1	0	1
Other			
Cellulitis	1	1	0
Delirium	2	2	0

**Table 3:** Comparison of features between patients with and without postoperative complications among 25 patients ≥ 85 years treated with curative surgery.

Variables	With Postoperative Complication (n = 8)	Without Postoperative Complication (n = 17)	*p*-Value
Age (years, mean)	89.4	88.2	0.025
Female	6 (75%)	10 (59%)	0.49
PS ≥ 2	5 (63%)	2 (12%)	0.017
BMI (kg/m^2^, mean)	20.1	21.8	0.51
ACCI ≥ 9	4 (50%)	2 (12%)	0.059
Extremity tumor	2 (25%)	14 (82%)	0.010
Diagnosis			0.095
UPS	5	7	
MFS	0	7	
DDLPS	0	0	
Others ^a^	3	3	
High-grade tumor	6 (75%)	14 (82%)	1.00
Size (cm, mean)	12.8	8.3	0.025
Unplanned excision	1 (13%)	6 (35%)	0.36
ASA ≥ Ⅲ	3 (38%)	3 (18%)	0.32
R1/R2 margin	2 (25%)	3 (18%)	1.00
Preoperative RT	1 (13%)	3 (18%)	1.00
Length of postoperative stay, days, mean	43.8	32.7	0.21

*PS*, performance status; *BMI*, body mass index; *ACCI*, age-adjusted Charlson comorbidity index; *UPS*, undifferentiated pleomorphic sarcoma; *MFS*, myxofibrosarcoma; *DDLPS*, dedifferentiated liposarcoma; *ASA*, American Society of Anesthesiologists; *RT*, radiotherapy. ^a^: Other diagnoses included pleomorphic liposarcoma (n = 1), extra-skeletal chondromyxoid sarcoma (n = 1), leiomyosarcoma (n = 1), fibrosarcoma (n = 1), malignant tenosynovial giant cell tumor (n = 1), and unknown (n = 1).

**Table 4:** Postoperative stay and complications according to soft tissue reconstruction in 25 patients undergoing curative surgery.

Reconstruction Method	Patients, n (%)	Mean Postoperative Stay, Days	Complication Rate, n (%)
Primary closure	9 (36%)	24.9	2 (22%)
Skin grafting	5 (20%)	33.4	1 (20%)
Local skin flap	3 (12%)	42.3	0 (0%)
Pedicled flap	6 (24%)	53.3	5 (83%)
Free flap	2 (8%)	30.0	0 (0%)

## Data Availability

The datasets used and analyzed during this study are available from the corresponding author upon reasonable request.
